# Hyperuricemia and the Risk of Heart Failure: Pathophysiology and Therapeutic Implications

**DOI:** 10.3389/fendo.2021.770815

**Published:** 2021-11-12

**Authors:** Ke Si, Chijing Wei, Lili Xu, Yue Zhou, Wenshan Lv, Bingzi Dong, Zhongchao Wang, Yajing Huang, Yangang Wang, Ying Chen

**Affiliations:** Department of Endocrinology, Affiliated Hospital of Qingdao University, Qingdao, China

**Keywords:** hyperuricemia, heart failure, cardiovascular disease, pathophysiology, treatment

## Abstract

The association between hyperuricemia and cardiovascular disease (CVD) has been reported and studied in the past two decades. Xanthine oxidase (XO) induced uric acid (UA) serves as a risk factor and has the independent prognostic and functional impact of heart failure (HF), but whether it plays a positive role in the pathogenesis of HF has remained unclear. Growing evidence suggest the up-regulated XO avtivity and increased production of free oxygen radical (ROS) correspondingly are the core pathogenesis of HF with hyperuricemia, which results in a whole cluster of pathophysiologic cardiovascular effects such as oxidative stress, endothelial dysfunction, vascular inflammation, left ventricular (LV) dysfunction as well as insulin resistance (IR). The use of XO inhibition represents a promising therapeutic choice in patients with HF due to its dual effect of lowering serum UA levels as well as reducing ROS production. This review will discuss the pathophysiologic mechanisms of hyperuricemia with HF, the targeted therapeutic interventions of UA lowering therapies (ULT) with XO inhibition and mechanism underlying beneficial effects of ULT. In addition, the review also summarizes current evidence on the role of ULT in HF and compares CV risk between allopurinol and febuxostat for practical and clinical purposes. Guidelines and implementation of CV risk management in daily practice will be discussed as well.

## Introduction

Hyperuricemia is commonly defined as a serum UA concentration > 6.8 mg/dL, resulting predominantly from reduced renal excretion of uric acid (UA) (1). In recent years, the prevalence of hyperuricemia has been increasing worldwide and was seen in 14.6% of the US population (estimated 32.5 million individuals) in 2015. A number of epidemiological studies have shown that hyperuricemia is associated with the development of cardiovascular disease (CVD), chronic kidney disease (CKD), diabetes and metabolic diseases. Among them, the relationship between hyperuricaemia and heart failure (HF) has gained much attention for many years (2, 3).

As a systemic disease, HF represents hemodynamic failure and neuroendocrine activation of multiple other organs and systems. HF represents a growing public health burden with mortality rates and prevalence expecting to increase by 46% from 2012 to 2030, resulting in more than 8 million US adults with HF (4). In developed countries, it is reported that HF is the leading cause for non-elective hospitalizations for patients over 65 (5).

To date, a clear pathophysiological link between hyperuricemia and HF has yet to be confirmed. However, UA has been related to many of the established risk factors for HF, implying that hyperuricemia may play a vital role in HF. Findings from experimental studies indicated that the presence of hyperuricemia independently predicted the development of HF, including in individuals with normal cardic function. A systematic review and meta-analysis that reported HF morbidity and outcomes of adult patients found that hyperuricemia was associated with an increased risk of incident HF [hazard ratio (HR) 1.65, 95% confidence interval (CI) 1.41—1.94], and for every 1 mg/dL increased in UA, the odds of development of HF increased by 19% (6). Studies have demonstrated that hyperuricemia could be not only a risk factor but a strong and independent predictor of adverse outcomes of HF. Yuta et.al (7) had a finding regarding hyperuricemia and poor outcomes in 516 consecutive hospitalized HFpEF patients with decompensated HF, showing that hyperuricemia was significantly associated with increased incidence of all-cause death (p=0.016). Serum UA is a simple and inexpensive laboratory measurement with a wide range of clinical applications. Studies specifically found that UA could be a better predictor in HF of disease progression and impaired prognosis than BNP, but the threshold of predicting poor outcome has not been standardized yet. What’s more, the debate is ongoing whether UA is merely a marker of poor prognosis or an active participant in pathogenesis of HF. Although strong evidence is emerging to prove a causal relationship between hyperuricemia and HF, most Mendelian randomization studies suggest serum urate is noncausal for comorbid traits (8). Additionally, asymptomatic hyperuricemia has been reported to increase a significant risk for cardiometabolic disorders (9). Serum UA levels can provide prognostic information alone or can be used in combination with other indicators of cardiac function, including left ventricular EF%. There are two major HF phenotypes, reduced versus preserved ejection fraction heart failure (HFrEF, HFpEF). Alberto et.alproved that in patients with HFrEF and HFpEF, hyperuricemia was related to the primary out of hospitalization or death, and the prevalence of hyperuricemia and the strength of its relationship with the primary outcome was greater in those with HFpEF. A study of elderly hypertensive outpatients found a strong inverse relationship between SUA and EF% for patients with mild to moderate HF. Moreover, elevated serum UA levels are independent predictor of mortality in patients with moderate-to-severe HF (10). The relationship between UA and EF% is independent of kidney function and diuretic usage, which excludes the possibility that the impaired renal excretion of UA is responsible for the association between hyperuricaemia and left ventricular dysfunction, even if this assumption requires further confirmation (e.g.by measures of 24-h uric acid excretion). Recent clinical evidence supported an expanded role for xanthine oxidase (XO) pathway in the pathogenesis of HF, as up-regulated activity of XO and increased reactive oxygen species (ROS) might lead to oxidative stress, endothelial dysfunction, vascular inflammation, etc, having detrimental effects on HF. In addition, as a UA lowering drug, allopurinol was reported to ameliorate outcomes in HF patients and become a marker of improved survival in the Seattle Heart Failure Model. These findings stimulated a growing research interest on the potential benefits of UA lowering therapies (ULT) and mechanisms underlying their effects on HF. In this review, we will discuss the underlying pathophysiology of hyperuricemia involved in the pathogenesis of HF. In particular, we will pay special attention to the potential effects and clinical implications of ULT on the progression of HF by reviewing most of available data on the medications related to hyperuricemia management. Finally, we will consider topics that need further research with the aim to decrease the HF burden of patients with hyperuricemia.

## Hyperuricemia: Pathophysiology in HF

**Figure 1:**
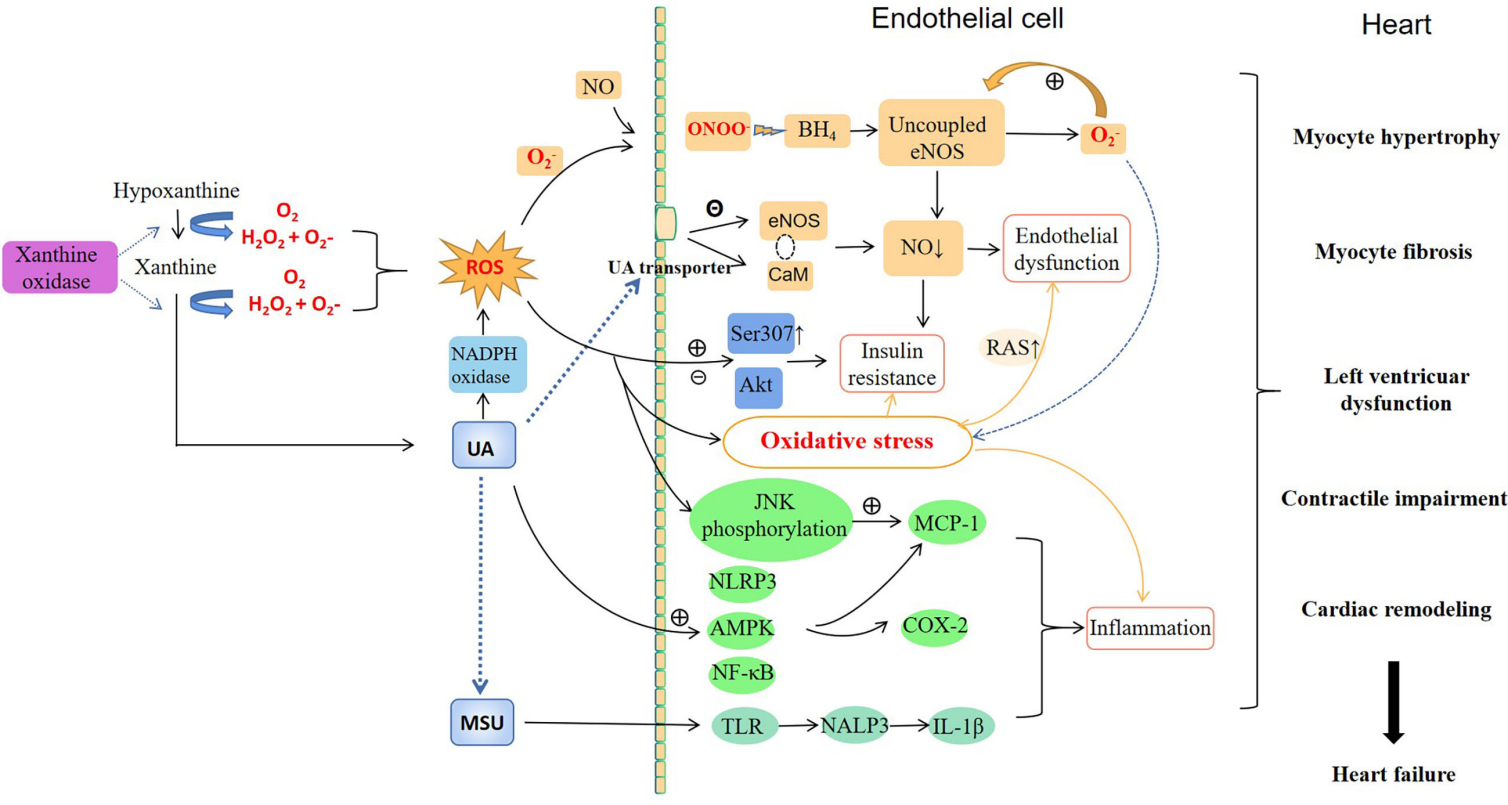
Potential mechanisms by which hyperuricemia might mediate HF.

Although the causal relationship between hyperuricemia and HF remains unknown, experimental and clinical studies have suggested hyperuricemia may be pathogenic and participates in the pathophysiology of HF by serving as a bridging mechanism mediating the deleterious effects on HF. **Figure 1** illustrates the possible pathophysiological mechanisms linking hyperuricemia and HF.

### Oxidative Stress

Up-regulated XO can contribute to the pathogenesis of HF through the oxidative stress induced by XO-derived UA and ROS. As an antioxidant, UA is capable of neutralizing dangerous pro-oxidants (11). However, evidence suggests that UA may function as a pro-oxidant in the hydrophobic intracellular environment, either by generating free radicals or by stimulating nicotinamide-adenine dinucleotide phosphate (NADPH) oxidase. But more data must be collected to confirm this theory. Certainly, UA can reflect the potential activity of XO (12). Different from UA, XO-derived ROS plays a greatly clear role in promoting oxidative stress in HF. There are various sources of ROS within the cells, such as free radical superoxide anion (O_2_^-^), hydrogen peroxide (H_2_O_2_), and peroxynitrite (ONOO^-^) (13). XO is the major CV sources of ROS in higher mammals that thought to have a prominent effect on cardiac function. The increased markers of oxidative stress have been observed in animal models of HF, which supports the theory that ROS may be the result of the progression of myocardial failure.

Increased ROS and UA production can lead to excess oxidative stress, protein and lipid peroxidation, DNA mutagenesis and eventually contribute to irreversible cardiomyocytes damage. Yu et.al (14) found a novel mechanism of UA-induced endothelial dysfunction-oxidative stress with an activation of the renin-angiontensin system (RAS) in human vascular endothelial cells. ROS interacts readily with endothelium-derived nitric oxide (NO) to produce ONOO^-^ (15), which decreases NO bioavailability (16) and starts a cascade of detrimental oxygen radical effects on endothelial cell, causing vascular endothelial dysfunction in HF. In addition, excess cardiac fibrosis is considered as an important detrimental factor of chronic heart failure (CHF). ROS plays a role in cardic fibrosis by inducing cardiac fibroblast proliferation and activating XO-mediated matrix metalloproteinases (MMP), leading to extracellular remodelling (17). James et.al (18) reported that ROS depressed Ca^2+^ accumulation by sarcoplasmatic reticulum (SR) and Ca^2+^ ATPase of SR, inducing a decrease in cardiac contractility. Allopurinol diminished the ROS effects on myofilament Ca^2+^ sensitivity, contributing to the improvement of LV contractile function and efficiency. What’s more, cardiomyocyte ROS and MMP activation may play a causative role in the myofibrillar degeneration, and are responsible for myosin and troponin degradation during ischemia/reperfusion injury of the heart, resulting in LV dysfunction (19). Accumulating data shows that this impaired oxidative metabolism is the core pathogenesis of HF with hyperuricemia, and it is implicated in the development of endothelial dysfunction, myocardial fibrosis, LV remodelling, and contractility impairment responsible for worse clinical status in patients with HF.

### Endothelial Dysfunction

Endothelial dysfunction can be defined as the condition that impairs the balance between endothelium-dependent vasodilation and constriction. NO is generated by endothelial NO synthase (eNOS), and reduced NO bioavailability contributes to endothelial dysfunction and oxidative stress, which is the key mechanism of CV risk and dysfunction. Experimental studies have indicated that UA absorbes into endothelial cells *via* UA transporters and induces oxidative stress, inflammation, or proliferation of vascular smooth muscle cells (VSMC), contributing to endothelial dysfunction through a reduction of endothelial NO bioavailability (20). Studies reported UA could attenuate eNOS activity and NO production or in human umbilical vein endothelial cells (HUVEC) by significantly decreasing the interaction between eNOS and calmodulin (CaM) or enhancing protein kinase C (PKC)-dependent eNOS phosphorylation (21, 22).

In environments of high ROS, O_2_^-^ reacts avidly with vascular NO to form ONOO^-^. Considered as a highly active oxygen radical, ONOO^-^ leads to lipid peroxidation and destroys endothelial membrane, accelerating the development of endothelial dysfunction (16). Moreover, ONOO^-^ can oxidize tetrahydrobiopterin, the vital eNOS cofactor, so that eNOS failures to produce NO but O_2_^-^. The process is referred to as eNOS uncoupling (23). O_2_^-^ can also induce endothelium injury directly and further promote eNOS uncoupling through a vicious cycle, contributing to ventricular remodeling and HF ultimately. Ajit ed.al (24) found that ROS regulated canonical Wnt signaling, inducing vascular endothelial dysfunction *via* redox regulatory protein p66(Shc)-regulated ROS.

### Vascular Inflammation

It is well documented that hyperuricemia induces vascular inflammation *via* multiple mechanisms including oxidative stress, VSMC proliferation, and endothelial cells injury. A finding has been shown that soluble UA could release chemokines and adhesion molecules and interfere with the formation of endothelial cell tubes in HUVECs in a dose-dependent way (25). Hui et.al (26)revealed that UA induced inflammation *via* Nod-Like Receptor Protein 3 (NLRP3)-inflammasome-mediated VSMC proliferation. AMP-activated protein kinase (AMPK) and nuclear factor-κB (NF-κB) are major pathways in mediating inflammatory response and participating in the expression of inflammatory factors induced by UA (27, 28). The inflammatory response caused by the deposition of monosodium urate (MSU) in the synovium can be recognized by Toll-like receptor (TLR), and then activates NACHT-PYD-containing protein 3 (NALP3) inflammasome. This is the most important mechanism of IL-1βsecretion. MSU-triggered neutrophils adhere to the endothelium, traverse through the vessel wall and reach the site of inflammation, contributing to a proinflammatory response by producing immune mediators (29).

Although ROS is essential for vascular homoeostasis, excessive ROS may cause vascular damage. UA and MSU can mediate the generation of ROS, which induces inflammation and promotes the progression of HF. ROS production is observed in MSU treated macrophages and the use of antioxidants or knockdown of NAPDH subunit expressions can inhibit MSU-induced inflammasome activation (30, 31). ROS induces the phosphorylation of c-Jun N-terminal kinases (JNK), contributing to the production of monocyte chemotactic protein-1 (MCP-1) in macrophages (32). Therefore, inflammation from soluble UA, MSU and ROS might together contribute to the progression of HF, but it is often difficult to distinguish which mechanism acts first in CVD.

### Left Ventricular Dysfunction

Decompensation of cardic function can be observed in patients with HF, leading to LV dilation, hypertrophy, and myocardial fibrosis, further results in remodeling of the ventricular structure. LV dysfunction contributes to reduced ventricular compliance and decreased systolic function, consequently causes HF. The mechanisms of hyperuricemia causing LV dysfunction have not been definitely investigated. Several studies show elevated UA levels might result in the echocardiographic abnormalities related to HF by affecting endothelial function and inflammation. UA has been reported to inhibit both NO production by vascular endothelial cells and their proliferation and migration, prompting LV development (33). Microvascular dysfunction caused by inflammation can result in deposition of collagen with subsequent reduced ability of the myocardium to contract and relax, developing into HF with preserved ejection fraction (34, 35). In addition, recent studies have shown that hyperuricemia significantly induces cardiomyocyte apoptosis, interstitial fibrosis, diastolic dysfunction and ventricular remodeling through activation of calpain-1 and endoplasmic reticulum (ER) stress (36) or a ROS-dependent endothelin-1 (ET-1) pathway (37), accelerating the occurrence and deterioration of CVD.

### Insulin Resistance

Studies have found myocardial insulin mediates energy uptake by increasing the absorption of glucose and plays an essential role in protection against post-ischemic HF, while IR promotes the progression of HF. The causal relationship between hyperuricemia and IR has not been clearly determined and is under investigation. It was reported that increased UA concentration could reduce NO levels and further reduce the insulin sensitivity. Other reports showed that UA induced IR by increasing tissue NADPH oxidase or hs-CRP level, the latter was found to be an independent predictor of homeostatic model assesssment-insulin resistance (38). Oxidative stress induced by ROS may play a causal role in IR-related CV complications (39). Li Zhi et.al (40) reported hyperuricemia could increase ROS production and inhibit insulin-induced glucose uptake in H9c2 and primary cardiomyocytes. N-acetyl-L-cysteine (ROS scavenger) pretreatment could reverse the inhibitory effect. The mechanism may be that hyperuricemia increases phospho-IR substrate 1 (Ser307) and inhibits phospho-protein kinase B (Akt) response to insulin in myocardial tissues. It is reported that ULT in patients with hyperuricemia can improve IR. A double-blind crossover trial that randomly assigned patients to benzbromarone or placebo indicated that patients with hyperuricemia and HF showed an improvement in IR index (placebo, 5.4+/-2.6; benzbromarone, 3.0+/-1.7; P<0.05) (41). However, whether hyperuricemia has a causal relationship with IR and diabetes remains controversial and the mechanisms of myocardial IR induced by UA have not been fully elucidated. Nonetheless, it still can be a novel potential mechanism of CVD related to hyperuricemic.

## Therapeutic Implications

The important contribution of hyperuricemia to the pathophysiology of HF may indicate that therapeutic strategies aimed at ULT may beneficially influence the course of the disease. In the next paragraphs we will explore the potential mechanisms of ULT on HF, discuss CV effects of drugs for hyperuricemia treatment and further compare whether allopurinol or febuxostat is more effective in treating HF.

### Mechanism Underlying Beneficial Effects of ULT

Currently, two potent classes of ULT medications are commonly used in clinical practice: XOI (e.g., allopurinol, febuxostat) and increasing UA excretion drugs (e.g., benzbromarone, probenecid). Studies have shown that ULT is associated with reduced risk of HF in hyperuricemic patients but the potential mechanisms remain uncertain. Moreover, it has not yet been definitely proved whether the cardioprotective effects of ULT are due to XO inhibition or UA reduction.

Actually, XO is a critical source of ROS that accounts for a range of detrimental processes in the pathophysiology of HF. XO inhibition treatment from ULT shows a beneficial effect on the outcomes in HF patients. Allopurinol has been reported to improve myocardial oxidative stress and attenuate cardiac fibrosis in cardiac diastolic dysfunction (42). George et.al (43) showed a dose-response curve for allopurinol and its effect on endothelial function that allopurinol significantly increased forearm blood flow response to acetylcholine. Febuxostat has been shown to control the formation of ROS and act against vascular inflammation promoted by oxidative stress (43). However, benzbromarone was reported to have no influence on BNP levels, NYHA functional class, or LV ejection fraction (LVEF) (42). The results demonstrated that UA lowering without XO inhibition might not improve hemodynamic impairment in pathophysiology of HF. What’s more, several studies have illustrated the effect of XO inhibition on improved LV ejection fraction, cardiac remodeling, and peripheral perfusion (44, 45). Recently, studies have demonstrated that SGLT2i can dramatically improve clinical outcomes in diabetes, especially HF and progression of kidney disease. Factors that may contribute to these findings include: (1) improved glycemic control, (2) reduced serum UA levels, (3) reduction in all-cause mortality, CV mortality and improving HF (46, 47). A meta-analysis of randomized controlled trials showed SGLT2i significantly reduced SUA levels compared to controls [total weighted average difference (WMD) -37.73μmol/L, 95% CI (-40.591, -34)] (46, 47). Serum UA decreased in SGLT2i users owing to the increased urinary excretion rate of UA, which is due to the inhibition of UA reabsorption mediated by the effect of the drug on the GLUT9, located at the collecting duct of the renal tubule (48). Therefore, SGLT2i has great benefits in reducing the risk of CV events in T2DM patients with hyperuricemia.

Accumulating evidence has suggested that blocking ROS accumulation may become a promising new treatment option for hyperuricemia. However, current studies confirm that ULT benefits young hypertensive patients, but the effect on HF has shown contradictory clinical outcomes. A network meta-analysis suggested that allopurinol therapy did not have a significantly low risk of mortality in terms of HF but might offset the adverse effects associated with long-term hyperuricemia in patients with HF (49). Givertz et al. found that after treatment of HF patients with allopurinol and placebo, There was no significant difference in changes in clinical status, 6-minute walk distances, and LVEF between two groups at 24 weeks, which may result from the study duration being not long enough to observe the benefits of XO inhibition (50). There has been no high quality RCT comparing allopurinol with placebo on clinical CV events. Therefore, an appropriate methodological approach is needed to evaluate the efficacy of XO inhibition and give a better description of the characteristics of HF patients. A longitudinal cohort in Taiwan found that serum UA ≥8 or <4 mg/dL could independently predict the elderly > 65 years with higher all-cause and CVD-related mortality (51). And when serum UA <4 mg/dL, the risks of mortality increased as serum UA levels decreased (52). High doses of allopurinol may have association with loss of CV protection (53). These results question the hypothesis that “the lower is better” regarding serum UA levels. U or J shaped association between serum urate and CV adverse outcomes was reported by some observational studies (51, 52). This may be due to the fact that UA is an important antioxidant, and low serum UA level may represent a decrease in total antioxidant capacity, which becomes an incentive for increasing CV risk. As have discussed above, there is still a controversy about ULT on CV outcomes in hyperuricemic patients with HF. Conversely, With regard to the impact of HF on ULT, furosemide prescribed for patients with HF may eliminate the inhibitory effect of allopurinol on XO (54). Therefore, the minimal effective dose of diuretics should be kept in order to decrease the risk of CVD. Underlying mechanisms and beneficial effects of ULT are needed to be further explore and prove in future studies. Besides, CV safety trials are required before guidelines recommend reducing UA below a certain threshold.

### Comparative CV Risk Between Allopurinol and Febuxostat

Studies reporting on the relationship between ULT and CV risk have demonstrated conflicting results (**Table 1**). To date, most studies have been limited to allopurinol until the cardiovascular safety of febuxostat and allopurinol in patients with gout and cardiovascular morbidities (CARES) trial was initiated (58). Higher all-cause and CV mortality were found in patients with febuxostat gradually. Therefore, it is necessary to discuss and evaluate CV safety in allopurinol versus febuxostat.

**Table 1:** Studies to assess or compare the effect of XO inhibitors in CVD.

Study	Study design	Population	Mean follow-up	Treatment	Results	CV risk by treatment
**Comparison between a XO inhibitor versus placebo**						
Awsan Noman et al (55), (UK)	Randomized, double-blind, placebo-controlled, crossover study	Chronic stable angina	12-weeks	Allopurinol	Allopurinol prolonged the time to the total exercise time (58s median increase, p=0.0003), the time to angina (38s median increase, p=0.001), and ST-segment depression (43s median increase, p=0.0002)	Reduced
Li Wei et al (56), (Scotland)	Cohort study	Elderly (≥60 years old)	5-years	Allopurinol	High-dose (≥300 mg) allopurinol had reduced risk of CV events (adjusted HR 0.69,95%CI 0.50–0.94) and mortality (adjusted HR 0.75,95% CI 0.59–0.94)	Reduced
Lhanoo Gunawardhana et al (57), (USA)	Phase II, multicenter, placebo-controlled, double-blind proof-of- concept study	Gout	3-months	Febuxostat	Febuxostat lowered serum UA effectively and did not show an increased risk of CV complications	No difference
**Comparison between XO inhibitors**						
William B. White et al (58), (USA)	Multicenter, double-blind, noninferiority trial	Gout with CVD	32-months	Allopurinol *vs* Febuxostat	All-cause and CV mortality were higher in the febuxostat group than in the allopurinol group [HR for death from any cause, 1.22 (95% CI, 1.01 to 1.47); HR for CV death, 1.34 (95% CI, 1.03 to 1.73)].	Higher risk in febuxostat
Arrigo Francesco Giuseppe Cicero et al (59), (Italy)	Cohort study (prospective)	Elderly with CHF	5-years	Allopurinol *vs* Febuxostat	Febuxostat had a better CV outcome in patients treated with in comparison with allopurinol (The cumulative CV survival was 0.96 (95% CI 0.93–0.99) in febuxostat group and 0.89 (95% CI 0.84–0.93) in allopurinol group.	Lower risk in febuxostat
Isla S Mackenzie et al, (UK, Denmark (60), and Sweden)	Multicentre, prospective, open-label, non-inferiority trial	Elderly with Gout	4-years	Allopurinol *vs* Febuxostat	Febuxostat is non-inferior to allopurinol therapy about the primary cardiovascular endpoint, and it is not associated with an increased risk of death or serious adverse events compared with allopurinol.	No difference

XO, xanthine oxidase; CVD, cardio vascular disease; UA, urate acid; CHF, Congestive heart failure.

As a XO enzyme inhibitor, allopurinol becomes currently the accepted first-line treatment as ULT for hyperuricemia. However, observational studies on whether the use of allopurinol may be associated with improved CV outcomes are inconclusive. Study showed allopurinol could significantly lower LVEF and improved CFR (61). In an RCT of 65 patients with coronary disease, allopurinol markly prolonged the time to the total exercise time (58s median increase, p=0.0003), the time to angina (38s median increase, p=0.001), and ST-segment depression (43s median increase, p=0.0002) (55). The study by de Abajo et al. found that allopurinol appeared to increase CV protection with greater duration of treatment and higher dose (62). The finding is consistent with the results from Wei et al. who observed a dose dependency with reduced CV events and mortality in HF in high dose compared with low-dose group (HR=0.63; 0.44 to 0.91). Recently, A study in Taiwan found that CVD could increase the risk of allopurinol hypersensitivity (63).

Compared with allopurinol, febuxostat provides highly selective and effective inhibition of XO and has higher UA lowing activity. Clinical trials found that febuxostat may improve oxidative stress status or ameliorate inflammation of hemodialysis patients with endothelial dysfunction (64, 65). In a phase II, multicenter, placebo-controlled study of 189 patients with gout, febuxostat was well tolerated in patients with gout and did not show an increased risk of CV complications (57). However, the safety of febuxostat shows conflicting results. FDA adverse event reporting system (AERS) in US reported febuxostat-related CV thromboembolic events from the database (66). The CARES trial conducted by the US Food and Drug Administration (FDA) has observed that the major CV events of febuxostat group were similar to those associated with allopurinol treatment. However, all-cause and CV mortality were higher in the febuxostat group comparable to those of allopurinol [HR for death from any cause, 1.22 (95% CI, 1.01 to 1.47); HR for CV death, 1.34 (95% CI, 1.03 to 1.73)] (58). Therefore, CARES results do not support the use of febuxostat as first-line treatment in ULT. However, there are some uncertainties from the results of CARES, such as high discontinuation rate and loss (67). With regard to the results in CARES, Montenegro et.al (68) proposed a possible explanation that febuxostat was more effective in blocking the reduction of XO-dependent nitrite levels, as compared with allopurinol, which might reduce beneficial effects of NO in CV homeostasis. Of note, there is no evidence to suggest that febuxostat is linked with greater CV risk than no XOI treatment. Nevertheless, the FDA has issued a black-box warning to restrict the use of febuxostat to gout patients who have failed or cannot tolerate maximum dose of allopurinol (69). However, subsequent studies still have shown inconsistent outcomes. A study showed that febuxostat might favorably affect CV mortality compared with allopurinol in elderly patients with mild-to-moderate HF (59). Recently, a randomized, blinded-endpoint trial in patients with gout in the UK, Denmark, and Sweden reported the long-term use of febuxostat didn’t contribute to an increased risk of death or serious adverse outcomes compared with allopurinol (60). In HFrEF patients with elevated UA levels, XOI with allopurinol did not improve clinical status, exercise capacity, quality of life, or LVEF at 24 weeks (50).To date, the exact effect on the CV risk between allopurinol and febuxostat has not been definitely proved and the mechanisms underlying these findings remain unanswered. More evidence is required to evaluate the CV risk of these drugs and guide clinical use in hyperuricemia.

## Management of CV Risk

UA production and metabolism are complex processes and many enzymes are involved in the conversion of the adenine and guanine to UA. Initially, adenosine monophosphate (AMP) and guanine monophosphate (GMP) are converted into inosine and guanosine by deaminase and nucleotidase respectively, and are further converted into the purine bases hypoxanthine and guanine by purine nucleoside phosphorylase (PNP), which are finally oxidized by XO to form the final product UA (70). Therefore, XOI have thus been proposed as a strategy for reducing UA. In addition, for hyperuricemic patients combined with CV risk factors, not only medication but also life management should be implemented simultaneously. The European League Against Rheumatism (EULAR) guideline recommends that patients with gout should avoid excessive intake of meat and seafood, alcohol, and soft drinks that contain fructose. It has been reported that the metabolism of fructose stimulates the production of UA, because transient ATP consumption is usually accompanied by the production of AMP and stimulates the AMP deaminase (AMPD) to catalyze the degradation of AMP to inosine monophosphate and increase the degradation of purines (71). However, severe purine restriction can contribute to increased consumption of carbohydrates and saturated fats, which in turn lead to IR, triglycerides, and LDL cholesterol (72). Despite smoking has been found to be negatively correlated with gout recently (73), smoking cessation still be encouraged in the EULAR recommendations. It is reported that weight loss achieved by lifestyle changes reduced serum UA levels by 18% with a decrease in XOD activity (74). Furthermore, regular physical activity produces many cardioprotective effects including beneficial physiologic remodeling of the heart.

A retrospective matched cohort study found patients with gout had already an increased CV risk profile at the date of their incident diagnosis, and they were more likely to have prior CVD (75), which provides strong evidence for treatment of gout in primary care guidelines on CV risk management (75). International studies suggest that more than half of adverse outcomes can be prevented in patients with CVD risk by making sure everyone take aspirin, stay smoke free and control their blood pressure and lipid levels. These data indicate that all patients with gout are at high risk for CVD, and that screening and management of CVD risk may achieve a high therapeutic effect (76). Although there are guidelines for the administration of CV risk, management in clinical practice is often difficult due to a poor adherence to management in gout and therapies. Recently, among patients attending secondary care gout clinics in New Zealand, we found only 50% of eligible patients received aspirin treatment, 64% on β-blockersand and 53% on a statin (76). Nevertheless, a CVD care programme in New Zealand showed a successful implementation of CVD risk management. Patients with a 5-year CVD risk >10% were offered a single intensive nurse-led intervention session and the great improvements were observed in blood pressure, prescriptions of aspirin and statins, and uptake of nicotine replacement products (77). Therefore, formal CVD risk assessments, more in-depth interventions and community long-term care support networks are necessary for hyperuricemic patients with CV risk. Moreover, we should not forget that patients often suffer from other comorbidities as well, and optimal preventive treatment requires to pay attention to these comorbidities. In the seventh Korean National Health and Nutrition Examination Survey from 2016 to 2017, there was an approximate U-shaped association between serum UA levels and 10-year CVD risk scores in males and the risk of CVD was the lowest when the serum UA level was 6.9 mg/dL. An approximately J-type association could be found in women (78). Therefore, it is necessary to appropriately manage UA levels in high-risk groups to reduce the risk of CVD. In a prospective cohort of 1193 patients with gout, serum UA ≥0.36 mmol/L was associated with increased overall mortality (HR=2.33, 95% CI 1.60 to 3.41) and CV-related mortality (HR= 2.05, 95% CI 1.21 to 3.45) (79). Maintaining serum UA <360μmol/L in a long term should be the main goal for these high-risk patients to reduce CV events and prolong patient survival. The EULAR guideline recommends that serum UA levels should be monitored at <360 mmol/L and a lower UA target (300mmol/L) is recommended for patients with severe gout (80). For asymptomatic hyperuricemia patients, Multidisciplinary consensus in Taiwan suggests they needn’t immediate ULT, potential causes of hyperuricemia should be identified and appropriately dealt with, especially in diseases that may increase CV risks.

## Conclusions and Future Research

In the past two decades, a compelling body of evidence including both experimental and clinical has emerged, which directly links hyperuricemia with the development and progression of HF. The pathophysiologic mechanisms of hyperuricemia with HF is being recently explored by the new data from experimental, epidemiological and clinical intervention trials (**Table 2**). This review suggests that the up-regulated XO avtivity and increased production of ROS correspondingly are the core pathogenesis of HF with hyperuricemia, which results in a whole cluster of pathophysiologic CV effects. Therefore, XO itself may serve as a novel and promising therapeutic target and XO inhibition may potentially lead to better clinical outcomes in HF. To date, there is no RCT that has compared XOIs with uricosurics on the clinical CV events so that large trials are warranted to demonstrate which medication is better. From a clinical perspective, clinicians need to identify the threshold of UA where ULT can effectively improve the adverse outcomes of HF without increasing the mortality. In the future, larger studies are conducted to determine whether ULT can further improve the clinical prognosis of the heterogeneous population of HF patients and analyze the sensitivity of different types of HF such as HFpEF and HFrEF to XO inhibition, thus carrying out targeted and individualized treatment. Although additional studies are needed to determine the threshold of UA for treatment initiation and to confirm optimal target levels, we believe that there is sufficient evidence to recommend routine screening for hyperuricemia in patients with HF as part of clinical practice and consider initiation of ULT among those who are hyperuricemic with evidence of deteriorating cardic function, unless there are specific contraindications.

**Table 2:** Pathophysiologic mechanisms of hyperuricemia in HF and potential effects of ULT.

Pathophysiological mechanisms	UA	ROS	Mechanism underlying beneficial effects of ULT
Oxidative stress	a. UA functions as a pro-oxidant in the hydrophobic intracellular environment (by generating ROS or stimulating NADPH oxidase) b. UA induces endothelial dysfunction-oxidative stress with an activation of the RAS	a. ROS interacts with NO to produce ONOO^-^ and starts detrimental oxygen radical effects on endothelial cell b. ROS induces cardiac fibroblast proliferation and activates MMP and leads to cardic fibrosis and extracellular remodelling c. ROS depresses Ca^2+^ accumulation and Ca^2+^ ATPase of SR, and decreases cardiac contractility	Allopurinol has been reported to improve myocardial oxidative stress and attenuate cardiac fibrosis in cardiac diastolic dysfunction (42)
Endothelial dysfunction	a. UA induces oxidative stress, inflammation, or proliferation of VSMC, and reduces endothelial NO bioavailability b. UA attenuates eNOS activity and NO production or decreasing the interaction between eNOS and CaM or enhancing PKC-dependent eNOS phosphorylation	a. ROS-reduced ONOO^-^ leads to lipid peroxidation and destroys endothelial membrane b. ONOO^-^ causes eNOS uncoupling c. O_2_^-^ induces endothelium injury directly and further promotes eNOS uncoupling d. ROS regulates canonical Wnt signaling and induces vascular endothelial dysfunction	Allopurinol had effects on endothelial function that significantly increased forearm blood flow response to acetylcholine (43)
Vascular inflammation	a. UA induces inflammation *via* NLRP3-inflammasome-mediated VSMC proliferation or AMPK and NF-κB signal pathways b. MSU activates NALP3 inflammasome and secrets IL-1β	ROS induces the phosphorylation of JNK, and contributes to the production of MCP-1 in macrophages	Febuxostat has been shown to control the formation of ROS and act against vascular inflammation promoted by oxidative stress (43)
LV dysfunction	a. UA-induced inflammation can reduce ability of the myocardium to contract and relax b. UA activates calpain-1 and ER stress and induces cardiomyocyte apoptosis, interstitial fibrosis and diastolic dysfunction	ROS leads to ventricular remodeling through a ET-1 pathway	Allopurinol diminished the ROS effects on myofilament Ca^2+^ sensitivity, contributing to the improvement of LV contractile function and efficiency
IR	a. UA reduces NO bioavailability and generation of mitochondrial oxidative stress to result in IR b. UA inhibits insulin-induced glucose uptake in H9c2 and primary cardiomyocytes	ROS plays a causal role in IR-related CV complications	Benzbromarone improved in IR index (41)

XO, xanthine oxidase; UA, urate acid; ROS, reactive oxygen species; ULT, uric acid lowering therapies; RAS, renin-angiotensin system; NO, nitric oxide; ONOO-, peroxynitrite; O2-, superoxide anion; MMP, matrix metalloproteinases; SR, sarcoplasmatic reticulum; VSMC, Vascular Smooth Muscle Cells; eNOS, endothelial nitric oxide synthase; CaM, calmodulin; PKC, protein kinase C; NLRP3, Nod-Like Receptor Protein 3; AMPK, AMP-activated protein kinase; NF-κB, nuclear factor-κB; MSU, monosodium urate; NALP3, NACHT-PYD-containing protein 3; IL-1β, Interleukin-1β; JNK, c-Jun N-terminal kinases; MCP-1, monocyte chemoattractant protein-1; LV, left ventricular; ER, endoplasmic reticulum; ET-1, endothelin-1; IR, insulin.

## Author Contributions

KS contributed to the conception and the writing of the article. CW and LX performed the framework. WL, BD and YZ contributed to the English grammar. ZW and YH gave the constructive discussions to the article. YW and YC revised important intellectual content critically for important intellectual content. All authors contributed to the article and approved the submitted version.

## Conflict of Interest

The authors declare that the research was conducted in the absence of any commercial or financial relationships that could be construed as a potential conflict of interest.

## Publisher’s Note

All claims expressed in this article are solely those of the authors and do not necessarily represent those of their affiliated organizations, or those of the publisher, the editors and the reviewers. Any product that may be evaluated in this article, or claim that may be made by its manufacturer, is not guaranteed or endorsed by the publisher.
